# Low Anterior Resection Syndrome following Restorative Proctectomy for Rectal Cancer: Can the Surgeon Have Any Meaningful Impact?

**DOI:** 10.3390/cancers16132307

**Published:** 2024-06-24

**Authors:** Richard C. Garfinkle, Nicholas P. McKenna

**Affiliations:** Division of Colon and Rectal Surgery, Mayo Clinic, 200 1st Street SW, Rochester, MN 55905, USA; richard.garfinkle@mail.mcgill.ca

**Keywords:** Low Anterior Resection Syndrome, rectal cancer, surgery, risk factors

## Abstract

**Simple Summary:**

Rectal cancer is usually treated with surgery, radiation therapy, and chemotherapy. As survival and cure rates for rectal cancer continue to improve, there is increased awareness regarding the side effects that patients experience from the treatment they receive. The most common side effect of rectal surgery is altered bowel function, also known as “Low Anterior Resection Syndrome” (LARS). This results from missing part, or all, of the rectum, as well as the consequences of radiation therapy. However, there are several ways in which surgeons can minimize the effects of LARS after surgery and improve bowel function in their patients, even when the entire rectum needs to be removed. This article reviews the existing research on how surgical decisions and techniques can impact LARS following surgery.

**Abstract:**

Postoperative bowel dysfunction following restorative proctectomy, commonly referred to as Low Anterior Resection Syndrome (LARS), is a common long term sequela of rectal cancer treatment. While many of the established risk factors for LARS are non-modifiable, others may be well within the surgeon’s control. Several pre-, intra-, and postoperative decisions may have a significant impact on postoperative bowel function. Some of these factors include the extent of surgical resection, surgical approach, choice of anastomotic reconstruction, and use of fecal diversion. This review article summarizes the available evidence regarding how surgical decision-making can affect postoperative bowel function.

## 1. Introduction

The incidence of colorectal cancer continues to increase worldwide [1]. Rectal cancer comprises approximately one-third of new cases and is disproportionately arising in younger individuals [2]. While advances in the multimodal care of rectal cancer have improved oncologic outcomes, there is increased recognition of the long term sequelae and adverse effects of rectal cancer treatment. Rectal cancer survivors may face a variety of adverse effects, including emotional, psychological, financial, and physical burdens [3,4]. Postoperative bowel dysfunction, commonly referred to as Low Anterior Resection Syndrome (LARS), is likely the most prevalent and impactful in terms of overall health-related quality of life (QoL).

LARS is defined as disordered bowel function that develops following rectal resection, leading to a detriment in quality of life [5]. While the epidemiology of LARS was poorly understood for many years, the introduction of the LARS Score in 2012 provided researchers with a common outcome measure to better study the condition [6]. LARS is very common among rectal cancer survivors, with prevalences ranging from 50 to 70% in institutional series [5,7]. Patients with LARS may experience various symptoms, including fecal urgency and frequency, incontinence to liquid and stools, and clustering (or fragmentation) of bowel movements [6]. Although symptoms may improve somewhat over the first 6–12 months, LARS typically persists well into follow-up, with one recent study demonstrating no difference in LARS scores over two different follow-up points 5 years apart from one another [8]. Moreover, major LARS—defined as a LARS score ≥ 30—is significantly associated with worse overall quality of life (QoL), making it an important patient-centric outcome of rectal cancer treatment [7,9].

Many studies have attempted to estimate factors associated with the development of LARS. While the risk for LARS appears to be largely dictated by tumor height and the decision to administer neoadjuvant radiation therapy, other more modifiable factors may further impact postoperative bowel function [10]. Many of these factors include operative decisions related to surgical approach and bowel reconstruction, as well as postoperative outcomes such as anastomotic healing and fecal diversion. As such, the surgeon may have an active role to play in mitigating postoperative bowel dysfunction. The objectives of this manuscript are to review factors associated with LARS, with a special emphasis on those potentially modifiable factors within the surgeon’s control.

## 2. Pathophysiology of LARS

To better understand how intraoperative decisions and postoperative outcomes affect long term bowel function, it is helpful to review the pathophysiology of LARS (Table 1), which has previously been reviewed in a separate article by one of our co-authors (R.G.) [11]. The most obvious reason for LARS is anatomical in nature, resulting from the partial or complete loss of the rectum, depending on the level of transection. Reduced reservoir volume in the colonic conduit or decreased capacity of the rectum is thought to explain many of the symptoms of LARS, including urgency and incontinence. In addition to reduced or absent rectal capacity, rectal compliance is reduced after surgery [12,13], particularly in the setting of preoperative radiation therapy, which can cause pelvic fibrosis. A hypocompliant rectum results in decreased distensibility of the reservoir, exacerbating the capacitance issue. Conversely, colonic motility may increase after a restorative proctectomy. In animal studies, rectal resection was associated with an increased number and duration of colonic migrating motor complexes, which in humans are responsible for the propagation of stool to the rectum [14]. Surgical denervation of the colon, particularly the sympathetic nervous system, may also increase colonic motility, as parasympathetic innervation is left unopposed. Partial denervation of the colonic segment used as the conduit is thought to be a major cause of stool fragmentation due to the absence of negative feedback on the defecation reflex [15]. Finally, numerous iatrogenic insults may predispose to LARS: radiation therapy may cause toxicity to surrounding structures; pelvic surgery may inadvertently result in denervation of the residual rectum; and the introduction of endoanal stapling devices may cause structural damage to the sphincter muscles. In accordance with the above, the most consistently identified risk factors associated with the development of LARS include low-lying rectal tumors (requiring a low anastomosis and resection of the entire rectum) and neoadjuvant radiation therapy.

## 3. Can We Avoid a Total Mesorectal Excision?

As a surgeon, one way to avoid LARS in your patient may be to avoid a proctectomy altogether. In recent years, researchers have evaluated both nonoperative management (NOM) and local excision following neoadjuvant chemoradiation and/or neoadjuvant chemotherapy as an alternative to proctectomy. While the detailed oncologic outcomes and indications are outside the scope of this manuscript, the data suggest these strategies are effective in avoiding the morbidity of surgery without compromising oncologic outcomes. Several patient-reported outcomes are improved with organ preservation; however, given the lasting effects of radiation therapy in the pelvis, the benefits of organ perseveration in maintaining acceptable bowel function are less obvious. 

The largest study evaluating bowel function following a NOM strategy is from the Dutch Watch-and-Wait Consortium [16]. After 24 months of follow-up, 221 patients were surveyed. Major LARS was reported in approximately 25% of patients, and major incontinence (according to the Vaizey score) in 22%. While the incidence of major LARS is higher than what has been reported in the normative population [17,18], it is lower compared to patients who undergo low anterior resection. Even among elderly patients managed with NOM, the rate of major LARS was below 30% [19]. Similarly, a small European study reported major LARS in 33% of patients managed nonoperatively, with a trend towards worse bowel function when a higher radiation dose was administered to the anal sphincter complex [20]. Several small observational studies more directly compared bowel function with NOM as compared to restorative proctectomy. A matched-controlled study out of the Netherlands evaluated 41 patients managed with NOM and identified 41 control patients who underwent TME, matched for age, sex, tumor stage, and tumor height [21]. All patients were followed for a minimum of two years. NOM patients reported significantly fewer defecation problems (16.1% vs. 25.8%, *p* = 0.01) based on the EORTC-QLQ-C38, as well as reduced fecal incontinence according to the Vaizey score (28.8% vs. 39.8%, *p* = 0.02) and LARS score (26.0 vs. 35.5, *p* = 0.04). Another study using the Memorial Sloan Kettering Cancer Center Bowel Function Instrument reported better overall bowel function among NOM patients (76.0 vs. 55.0, *p* < 0.001), with the greatest differences being those on the urgency/soilage subscales [22].

Interestingly, local excision following chemoradiation appears to have a significant additional impact on postoperative bowel function, despite being a rectum-preserving strategy. In the Dutch Watch-and-Wait Consortium, 18 patients underwent local excision following failure of NOM. Among them, 10 (55.6%) reported major LARS after two years of follow-up [16]. In another study comparing 53 complete clinical responders managed by watch and wait to 29 near-complete responders managed with local excision, the latter was associated with worse Wexner incontinence scores and worse quality of life [23]. However, local excision still has benefits as compared to formal proctectomy. The recently published TREC trial, which randomized 55 patients to either radical resection or transanal endoscopic microsurgery following neoadjuvant treatment, reported improvements in bowel function and quality of life with local excision that were sustained at 36-month follow-up [24]. A similar multicenter cohort study of 121 patients reported fewer evacuatory symptoms and clustering in patients who underwent local excision compared to those who underwent TME and better bowel function scores using the Memorial Sloan-Kettering Cancer Center Bowel Function Instrument [25]. The NEO trial, a phase II single-arm trial that evaluated neoadjuvant chemotherapy (without radiation) and local excision in low-risk rectal cancer, evaluated bowel function in 58 patients. Only 14% of patients reported major LARS at 12-month follow-up, nearly identical to the rate of pre-treatment major LARS (10%) [26]. As such, it appears that the omission of radiation therapy may be even more impactful in preserving bowel function. 

When a formal proctectomy is indicated, the option of a partial (or tumor-specific) mesorectal excision (PME) may preserve postoperative bowel function as compared to a complete TME. In mid- to upper rectal cancers, a PME may be considered whereby the distal rectum and mesorectum are left in situ. Current guidelines support PME with a distal resection margin of 5 cm or greater, as distal tumoral spread past that point is seldom observed, particularly after neoadjuvant therapy [27]. A recent systematic review and meta-analysis demonstrated that PME was associated with less postoperative anastomotic leak (OR 0.42, 95% CI 0.27–0.67) and less major LARS (OR 0.34, 95% CI 0.28–0.40) as compared to complete TME [28]. Accordingly, TME vs. PME is one of the five variables included in the POLARS (Pre-Operative Low Anterior Resection Syndrome) risk score [29]. Given the oncologic equivalence, surgeons should strongly consider performing a PME when oncologically appropriate. 

The multidisciplinary care of rectal cancer is rapidly evolving, and new neoadjuvant treatment paradigms may further impact long term bowel function. Total neoadjuvant therapy, which includes the delivery of preoperative systemic chemotherapy either prior to or following standard chemoradiation, has become the standard of care for locally advanced disease. Early data have demonstrated that the incorporation of chemotherapy preoperatively has drastically impacted bowel function [30]. The long term results of the RAPIDO trial demonstrated similar findings when short-course radiation therapy was used. Among nearly 500 patients who were randomized to short-course radiation therapy and consolidation chemotherapy versus chemoradiation alone, LARS scores were no different three years after surgery [31]. The omission of preoperative radiotherapy altogether, another evolving treatment paradigm, is likely to have a larger impact on bowel function. Several observational studies reported on the oncologic safety and feasibility of radiotherapy omission in select mid-upper rectal cancers without a threatened mesorectal fascia [32,33]. In a large study from Memorial Sloan Kettering Cancer Center that compared several different neoadjuvant treatment strategies, including chemotherapy alone, only exposure to radiation therapy was associated with worse bowel function [30]. However, in the recent PROSPECT trial that compared neoadjuvant chemotherapy alone (with selective consolidation chemoradiation in 9.1% of patients) to chemoradiation, bowel function measured with the Memorial Sloan Kettering Cancer Center Bowel Function Instrument at 12- and 24-months postoperatively was not significantly worse in the chemoradiation group [34]. Bowel function was only improved in the neoadjuvant chemotherapy alone group 1–2 weeks prior to surgery, which reflects the early toxicities of radiation therapy. Table 2 reports some examples of recent rectal cancer trials that included bowel function as an outcome. It will be interesting to follow how these results evolve over time as new treatment paradigms continue to emerge.

## 4. Surgical Approach

The surgical approach to proctectomy is another variable that may impact bowel dysfunction. Currently, there are four main approaches that surgeons can choose from based on their individual experience and skillset, as well as the particularities of the case: (1) open surgery; (2) laparoscopic surgery; (3) robotic surgery; and (4) transanal TME (TaTME). While the latter three approaches offer several advantages in terms of patient recovery and morbidity as compared to traditional open surgery [35,36], their impact on LARS is less clear. Proponents of minimally invasive surgery have hypothesized that laparoscopic or robotic approaches may improve postoperative bowel function due to improved visualization throughout the pelvic dissection and a better ability to preserve autonomic nerves. Given that open surgery is still very commonly employed for rectal cancer surgery, this question remains relevant.

In 2021, a systematic review and meta-analysis evaluated 32 publications, including 5565 patients [37]. The authors reported a lower pooled incidence of major LARS among robotic resections (21.7%) as compared to laparoscopic (33.3%) and open (46.5%) resections. However, 90% of the included patients underwent open surgery, while only 2.9% and 1.3% underwent laparoscopic or robotic-assisted surgery, respectively. Furthermore, of the 32 studies, only one presented any within-study comparison between two surgical approaches (open and laparoscopy), and no difference in LARS was demonstrated [38]. We have reviewed our own institutional data, where three approaches (open, laparoscopic, and robotic) are commonly used for rectal cancer surgery. Over a 10-year period, 514 patients underwent restorative proctectomy for rectal cancer, and no difference in major LARS was observed between open and minimally invasive cases (44.5% vs. 41.0%). On multivariable regression analysis, the surgical approach did not have any significant association with major LARS, even when restricting the cohort to low rectal cancer only [39].

Among the minimally invasive surgical approaches, only a few studies have compared bowel function outcomes directly with one another. A recently published propensity-score matched analysis of 342 patients compared LARS scores at 6, 12, and 18 months following robotic vs. laparoscopic restorative proctectomy. The authors reported a lower incidence of major LARS with robotic surgery, citing the three-dimensional visual field, flexible instrumentation, and tremor-free movements as possible reasons for a better autonomic nerve-sparing dissection [40]. However, these findings have not been consistently reported. The ROLARR study, which was a multicenter randomized controlled trial comparing laparoscopic and robotic rectal cancer surgery, provided follow-up data on bowel function among those who underwent a restorative procedure [41]. After a median follow-up of greater than two years, the authors reported no difference in major LARS between laparoscopic and robotic-assisted surgery (65.1% vs. 60.9%). TaTME represents another minimally invasive approach to proctectomy that can help secure a distal margin and provide access to a difficult pelvis by working from the bottom up. While it can be attractive in some senses, studies have mainly suggested equivalent or inferior postoperative bowel function with TaTME. Some concerns specifically related to TaTME and bowel function include prolonged insertion of the transanal platform (with subsequent sphincter damage) and the performance (typically) of a low, hand-sewn anastomosis. In a large propensity score-matched cohort study of laparoscopic and TaTME resections, TaTME was associated with higher mean LARS scores (30.6 vs. 25.4, *p* = 0.010), and more major LARS (65% vs. 42%, *p* = 0.013) [42]. In a subsequent meta-analysis of 20 studies, no significant difference in major LARS was identified (risk ratio for TaTME: 1.13, 95% CI 0.94–1.35) [43].

## 5. Reconstruction, Vessel Ligation, and Anastomotic Outcomes

Anastomotic reconstruction with a neorectal reservoir has long been proposed as a technique to counter the loss of rectal storage function following proctectomy. Based on the notion of the ileal-pouch anal anastomosis, the colonic J-pouch was first described in 1986 and gained popularity as a means of preserving postoperative bowel function [44]. Other anastomotic techniques for increasing reservoir capacity include side-to-end (“Baker”) anastomosis and transverse coloplasty. All three of these reconstruction options have been compared to a traditional straight end-to-end anastomosis with varying results. A recent meta-analysis from 2023 included 32 randomized controlled trials on the subject [45]. They stratified outcomes according to the time from surgery: short term (0–8 months), intermediate (8–18 months), and long term (>18 months). The study convincingly reported decreased short term stool frequency with all three of the reservoir options as compared to a straight anastomosis and less short term incontinence with colonic J-pouch only. However, the associations with improved bowel function decreased in the intermediate follow-up period and were no longer present on long term evaluation. This finding may be explained by the fact that straight anastomosis can dilate somewhat over time, resulting in similar reservoir capacity as compared to the other techniques. Furthermore, both side-to-end anastomosis and transverse coloplasty were associated with increased sensations of incomplete evacuation, particularly in the short term. Transverse coloplasty was also associated with an increased risk of anastomotic leak and stenosis, while the other reservoir options had a similar complication profile to a straight anastomosis. Based on the improved short term bowel function with no associated increase in evacuation problems, the authors recommended that the colonic J-pouch should be the preferred anastomotic reconstruction [45]. However, the fact that outcomes all converge in the long term may render the technique less appealing to those who do not routinely offer it.

An additional intraoperative consideration that may affect bowel function is the level of vascular ligation during rectal surgery. Historically, the inferior mesenteric artery (IMA) pedicle was ligated high (near the aorta) to achieve an adequate lymph node harvest and staging. However, recent meta-analyses have reported equivalent oncologic outcomes between high and low ligation of the vessel [46,47], and authors have suggested that functional outcomes should be the main consideration when deciding on the level of vascular ligation. High ligation of the vessel may decrease blood flow to the anastomosis and risk autonomic nerve damage around the root of the IMA. Among patients who have undergone a high ligation of the IMA, studies have demonstrated significantly longer colonic transit below the sigmoid colon, decreased propagated colonic contractions, and increased spastic minor contractions [48]. However, most studies to date evaluating the association between vascular ligation and postoperative bowel dysfunction have reported similar outcomes with the two approaches, including a randomized controlled trial of 100 patients, which was powered to this outcome [49,50]. It appears that a high-ligation technique likely has minimal to no impact on the development of LARS, and surgeons can continue to employ the approach they feel most comfortable with.

Finally, anastomotic leaks can be extremely detrimental to long term bowel function and have been repeatedly associated with LARS in observational studies [51,52,53]. Anastomotic leakage and any form of postoperative pelvic sepsis can result in a fibrotic pelvis and a hypocompliant neorectal reservoir. Studies have shown that clinically significant leaks are more strongly associated with LARS as compared to silent, asymptomatic leak. In a series of 135 patients who underwent restorative proctectomy, median LARS score (30 vs. 27, *p* = 0.02) and the proportion of patients with major LARS (44% vs. 17%, *p* = 0.004) were significantly higher among patients who had a symptomatic leak as compared to an asymptomatic leak [51]. Furthermore, both the the median LARS score and distribution of LARS score categories were similar among patients with an asymptomatic leak and those with no leak. In another series of 1099 patients, there was a strong association between anastomotic leak and major LARS (relative risk of 2.3, 95% CI 1.4–3.9). The authors also reported a particular association between anastomotic leakage and symptoms of fecal urgency as a predominant problem [53]. In a population-based study using UK-based data, anastomotic leaks again maintained a strong association with postoperative bowel dysfunction, defined in this study as medical visits related to bowel symptoms and bowel prescriptions [54]. In a recent randomized controlled trial that established sacral nerve modulation as a promising treatment strategy for patients with LARS, anastomotic leakage was strongly correlated with non-response to electrode implantation during the test phase, emphasizing its deleterious consequences on normal pelvic function [55]. While a comprehensive review of anastomotic techniques and mitigation strategies for anastomotic leaks is beyond the scope of this manuscript, it goes without saying that surgeons should do everything in their power to prevent this impactful complication.

## 6. Fecal Diversion

Most patients who undergo restorative proctectomy for rectal cancer will have a temporary diverting loop ileostomy constructed concomitantly. Fecal diversion has been shown to reduce the septic complications of anastomotic leaks and may decrease the incidence of leakage as well [56,57]. However, loop ileostomies are not free for the patient; in addition to requiring a second operation to restore intestinal continuity, they can be associated with other adverse events such as dehydration and renal failure. Furthermore, there appears to be an association between fecal diversion and increased risk for LARS.

Indeed, fecal diversion is one of the five variables included in the POLARS pre-operative risk score for LARS [29]. Several additional studies have demonstrated consistent associations between a history of fecal diversion and long term bowel dysfunction [58,59,60]. The challenge conceptually has been the confounding effects of low anastomotic height and radiation therapy, as patients with those two factors are most likely to undergo diversion due to a heightened risk of anastomotic leak. Nonetheless, the POLARS score and other groups implemented regression techniques to report the independent effect of fecal diversion. Furthermore, there is some biological plausibility to support how fecal diversion, irrespective of its link with higher risk anastomoses, may impact postoperative bowel function independently. With prolonged fecal diversion, the defunctioned bowel undergoes several structural and functional changes that may contribute to increased colon transit and worse function following reversal. Studies performed in animals have shown that defunctionalization of the bowel leads to atrophy of its villi and muscular layers [61]. In human studies, fecal diversion was associated with a significant loss of muscular contractility and atrophy of intestinal villi [62]. Researchers have also revealed lower concentrations of peptide YY secreted in the mucosa of the ileum and colon distal to a loop ileostomy, the function of which is to inhibit gastric motility and promote water and electrolyte absorption in the colon [63]. As such, the absorptive capacity of the colon is reduced following reconstruction of the bowel.

With this in mind, the surgeon may consider omitting fecal diversion in appropriate cases. There is a growing body of evidence for transanal drainage tubes as an alternative to a diverting loop ileostomy [64]. However, this concept hasn’t gained traction in North America yet. Furthermore, the consequences of a non-diverted anastomotic leak with the associated pelvic sepsis can be so devastating that bowel function likely should not be the principal motivation for avoiding a stoma. However, the duration of fecal diversion is very much within the surgeon’s control. Vogel et al. performed a systematic review and meta-analysis of 11 studies that reported on functional outcomes following ileostomy reversal and reported a significant association between time to ileostomy and major LARS [65]. A more recent study similarly identified a significant association with longer times to ileostomy closure and LARS, identifying a cut-off point of 128 days to predict LARS following reversal [66]. While there are certainly other factors to consider, ileostomy closure should ideally occur before the 3-month mark to minimize postoperative bowel dysfunction. In the era of total neoadjuvant therapy for rectal cancer, this should be increasingly feasible. 

## 7. Conclusions

LARS is a common long term sequela following restorative proctectomy for rectal cancer. While much of the risk for LARS is dictated by tumor height and the need for neoadjuvant radiation therapy, there are several other modifiable factors that all surgeons should consider in an attempt to mitigate postoperative bowel dysfunction. The principal considerations include: (1) the need for a full TME when organ-preserving strategies (including PME) are oncologically feasible; (2) the construction of a neorectal reservoir (e.g., colonic J-pouch) to help against early LARS symptoms; (3) minimizing anastomotic leakage; and (4) the need for, and duration of, temporary fecal diversion. Ultimately, bowel function is but one of several competing outcomes alongside postoperative recovery and oncologic outcomes, which is what renders these clinical decisions so challenging.

## Figures and Tables

**Table 1:** Pathophysiology of LARS and possible associated risk factors.

Pathophysiology	Risk Factor
Reduced reservoir	Low tumor/anastomotic height *** TME (vs. PME) *** End-to-end (straight) anastomosis **
Reduced compliance	Radiation therapy *** Anastomotic leak ***
Increased colonic motility	Fecal diversion ** Extremes of age (younger) ** High ligation of IMA *
Sphincter damage/dysfunction	Radiation therapy *** Extremes of age (older) ** Endoanal stapling devices * Iatrogenic (surgical) damage to nerves *

*** good evidence, ** moderate evidence, * weak/no evidence. TME = total mesorectal excision; PME = partial mesorectal excision; IMA = inferior mesenteric artery.

**Table 2:** Examples of recent trials in rectal cancer that reported on postoperative bowel function.

Study	Intervention	Bowel Function Measurement	Outcome
PROSPECT	Neoadjuvant chemotherapy with selective LCRT vs. LCRT	MSKCCBFI 12 and 24 months	No difference between treatment arms
RAPIDO	SCRT with consolidation chemotherapy vs. LCRT +/− adjuvant chemotherapy	LARS Score 3 years	No difference in major LARS (58.8% vs. 76.4%)
CAO/ARO/AIO-12	Induction chemotherapy before LCRT vs. LCRT with consolidation chemotherapy	WFIS 12, 24, and 36 months	No difference between treatment arms; only slight improvements at 24 and 36 months
FOWARC	Neoadjuvant chemotherapy vs. LCRT (with or without Oxaliplatin)	LARS Score Variable time from surgery	Decreased major LARS in chemotherapy alone arm (34.7% vs. 16.7%)
GRECCAR-4	Neoadjuvant chemotherapy +/− LCRT (if good response) vs. neoadjuvant chemotherapy with high/low intensity LCRT (if bad response)	LARS Score 1, 4, 8, 12, 24, 36, 48, and 60 months	No difference between all four treatment arms

LCRT = long-course radiation therapy; MSKCCBFI = Memorial Sloan Kettering Cancer Center Bowel Function Instrument; SCRT = short course radiation therapy; LARS = Low Anterior Resection Syndrome; WFIS = Wexner Fecal Incontinence Index.
